# A Modular Localization System as a Positioning Service for Road Transport

**DOI:** 10.3390/s141120274

**Published:** 2014-10-28

**Authors:** Peter Brida, Juraj Machaj, Jozef Benikovsky

**Affiliations:** Department of Telecommunications and Multimedia, Faculty of Electrical Engineering, University of Zilina, Univerzitna 1, Zilina 010 26, Slovakia; E-Mails: peter.brida@fel.uniza.sk (P.B.); jbenikovsky@avasoft.sk (J.B.)

**Keywords:** localization, modular localization system, indoor positioning, hybrid positioning, seamless positioning

## Abstract

In recent times smart devices have attracted a large number of users. Since many of these devices allow position estimation using Global Navigation Satellite Systems (GNSS) signals, a large number of location-based applications and services have emerged, especially in transport systems. However GNSS signals are affected by the environment and are not always present, especially in dense urban environment or indoors. In this work firstly a Modular Localization Algorithm is proposed to allow seamless switching between different positioning modules. This helps us develop a positioning system that is able to provide position estimates in both indoor and outdoor environments without any user interaction. Since the proposed system can run as a service on any smart device, it could allow users to navigate not only in outdoor environments, but also indoors, e.g., underground garages, tunnels *etc*. Secondly we present the proposal of a 2-phase map reduction algorithm which allows one to significantly reduce the complexity of position estimation processes in case that positioning is performed using a fingerprinting framework. The proposed 2-phase map reduction algorithm can also improve the accuracy of the position estimates by filtering out reference points that are far from the mobile device. Both algorithms were implemented into a positioning system and tested in real world conditions in both indoor and outdoor environments.

## 1. Introduction

In the recent years use of smart devices has increased significantly. Due to this, together with the fact that these devices have built-in Global Navigation Satellite Systems (GNSS) receivers, many location-based applications and services were created. The functions of many of these applications rely on the GNSS system because of its global availability. However these systems are highly reliable on Line of Sight (LoS) conditions, which are not the case in the urban environment. When the direct signal path between a transmitter (GNSS satellite) and a receiver (smart phone) is obstructed, the smart phone receives signals affected by multipath propagation and is not able to detect the direct signal. Another, even worse, condition is when the power of the received signal is lower than the minimum threshold at the GNSS receiver.

In both cases, localization accuracy can decrease significantly or in worst case the position of the device cannot be estimated at all. In the past decade many localization systems based on radio networks were proposed to deal with this problem and provide position estimates in environments where GNSS systems cannot be used, especially in indoor environments.

The most popular area for positioning systems and location based services (LBS) [1,2] is transport. With smart devices equipped with GNSS receivers, new possibilities for service providers were opened. Nowadays almost everyone owns a smart device and uses some basic LBS, like position estimation and navigation. However, based on previous analysis of the GNSS systems, these do not always provide position estimates which are accurate enough to provide good quality of service to the end user. To improve the performance of positioning systems, especially in areas where GNSS signal coverage is poor, alternative position estimates can be provided by positioning systems based on radio networks [3]. This can provide accurate position information even in indoor environments and thus could provide opportunities to provide new services e.g., navigation in underground parking lots and garages.

In this paper we will propose a modular positioning system, which at this time consists of three modules which can operate seamlessly in any environment covered with ubiquitously used radio networks, namely Global System for Mobile Communication (GSM) and Wireless Fidelity (Wi-Fi). The idea is to propose a positioning system that will be able to work in any environment from open outdoor areas, to dense urban areas and indoor environments. The main advantage of this system is the fact that there is no need to modify the smart phone device and no need to build up new infrastructure. The service provider only needs to set up a localization server, which will do the position estimation based on requests from the users. Another advantage is that it can be extended by adding new modules based on new technologies to provide more robust and reliable results, once the technology becomes available in the smart device.

The rest of the paper will be organized as follows: In Section 2 related work in the area of positioning in radio networks is described, while in Section 3 the proposed modular positioning system will be introduced. Section 4 will introduce testing scenarios and the achieved results will be shown and discussed in Section 5. Section 6 will conclude this paper and will provide some thoughts about the future developments of the proposed system.

## 2. Positioning in Wireless Networks

In this section related work in the area of positioning using wireless networks will be described. Many different positioning systems have been developed recently utilizing different wireless technologies, e.g., Ultra-wideband (UWB) [4], ZigBee [5], Bluetooth [6], Wi-Fi [7–11], GSM [12–15] *etc*. In the paper we will focus on positioning in Wi-Fi and GSM since these technologies are almost ubiquitously deployed in the urban and indoor environment, where GNSS positioning is not reliable enough.

### 2.1. Positioning in GSM Networks

Since the GSM network represents the basis of modern area-wide wireless communication infrastructure, this technology is ideal for use in wireless positioning. The basic method for positioning in the GSM network is Cell of Origin (CoO) where the position of a mobile device is given by the position of the Base Transceiver Station (BTS) with the highest signal power. Advanced positioning in the GSM networks can be performed based on three approaches:

- Distance-based positioning,
- Angle-based positioning,
- GSM fingerprinting.

In the distance-based positioning the basic assumption is that the positioning service provider knows the exact positions of the BTS in the radio network and their transmission powers. In such a case the distance between the BTS and a mobile device can be calculated from the measured Received Signal Strength (RSS) using a suitable signal propagation model [13].

On the other hand, in angle-based positioning the only required information is the position of the BTS. The resolution of the measured Angle of Arrival (AoA) depends on the antenna configuration [13]. This information can be further diluted due to Non-Line-of-Sight (NLoS) conditions and multipath propagation. Angle-based positioning together with Round Trip Time (RTT) measurements is commonly used in the Enhanced Cell of Origin (ECoO) [14] method, where position of the mobile device can be estimated within a given sector of area covered by the BTS.

The last and one of the most common ways to provide positioning service in GSM networks is to use a fingerprinting localization framework. In GSM fingerprinting [15] the position of a mobile device is estimated by comparison of measured RSS values from surrounding BTS with RSS values stored in the database at the localization server. A detailed description of the fingerprinting framework will be provided in the Section 2.3.

### 2.2. Positioning in Wi-Fi Networks

Nowadays the Wi-Fi network infrastructure is almost ubiquitous in dense urban areas and in indoor environments and all new smart devices have integrated Wi-Fi transmitters. This has led to the development of positioning systems based on Wi-Fi networks, mainly in indoor environments. In Wi-Fi networks, like GSM, positioning can be performed in different ways. Positioning systems based on Wi-Fi commonly utilize one of the following frameworks:

- Distance- or angle-based positioning (multilateration),
- Wi-Fi fingerprinting.

Other positioning techniques can also be used, e.g., Cell of Origin (CoO) and propagation modeling; however these methods are not very common [16]. Propagation modeling is actually a modification of the fingerprinting approach where a radio map database is created using propagation models. This helps to reduce the complexity of the calibration phase; however the accuracy of such a system is significantly lower.

Multilateration positioning basically utilizes estimation of the distance or angle between transmitter and receiver. In Wi-Fi networks AoA can be estimated only when Multiple-Input and Multiple-Output (MIMO) technology is used and LoS conditions are required [17]. Nowadays this is not the common case, therefore positioning using AoA cannot be implemented in Wi-Fi.

On the other hand distance between transmitter and receiver can be estimated by measuring RSS Time of Arrival (ToA) or RTT [18]. Both methods have their drawbacks when it comes to the accuracy of the distance estimate. In RTT measurements the accuracy is affected by latencies, which falsify outgoing and incoming time stamps [19]. Resulting delays may have typical variation of 5 μs which results in 1500 m error in the distance estimation. Another error in distance estimation can be caused by clock drift of the RTT observations [20]. When RSS is used to estimate the distance between transmitter and receiver, accuracy is given mainly by the propagation model used to compute the distance from RSS. Another challenge for RSS-based lateration is the high time-variability of signal strength caused by fading and multipath phenomena [21].

The most popular localization framework in Wi-Fi networks is empirical fingerprinting. Like in GSM networks, RSS from Access Points (APs) in the communication range is measured and used to estimate the position of mobile devices by comparison with the radio map database. The main advantage is that fingerprinting positioning seems to be more immune to multipath propagation phenomena. Another advantage in contrast to lateration positioning, is that there is no need to know the position of APs. It is also important to note that in Wi-Fi networks the RSS values are measured from beacon signals. Therefore RSS is not affected by the adaptive power regulation implemented in APs and device does not need to connect to the network. Thus APs from different providers and with different transmitting power settings can be used without any impact on the positioning system.

### 2.3. Fingerprinting Localization Framework

In this section fingerprinting positioning will be described in detail, since it is the most common and reliable positioning framework used in both GSM and Wi-Fi networks. Fingerprinting seems to achieve best accuracy in comparison to other positioning frameworks, especially in areas with strong multipath propagation and NLoS conditions. The operation of positioning systems based on fingerprinting frameworks can be divided into two phases—the calibration phase and the positioning phase [22].

#### 2.3.1. Calibration Phase

The calibration phase, sometimes also called offline phase, must be performed before the deployment of the positioning service. It represents a necessary step in the fingerprinting localization framework. During this phase a radio map database is created and stored in the database at the localization server.

**Figure 1.**
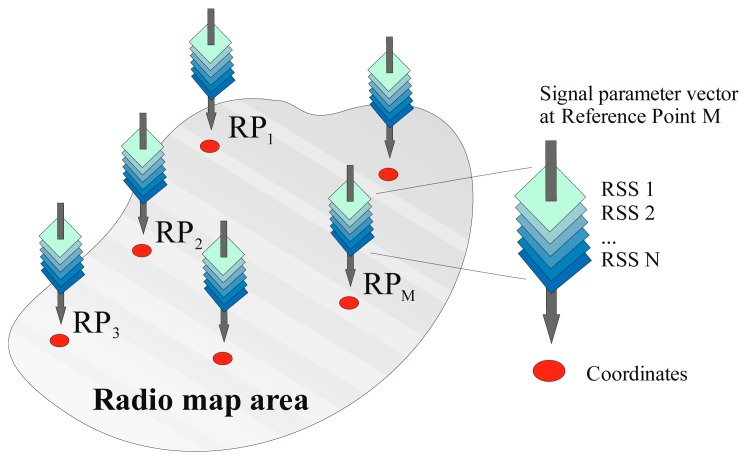
Radio map principle.

During the calibration phase the localization area is divided into small cells, where each cell is represented by a single reference point [9]. At each reference point RSS values from all APs within range are measured and stored in the radio map database, which is a collection of data vectors that can be described as:

(1) $$\begin{array}{cc} S_{j}=\left(\alpha_{1},\ldots ,\alpha_{N_{j}},c_{j},\theta_{j}\right) & j=1,2,\ldots ,M \end{array}$$

where *N_j_* is the number of APs heard at the *j*-th reference point, *M* is the number of reference points, *α_i_* are RSS values, *c_j_* represent coordinates of *j*-th reference point and parameter vector *θ_j_* can contain any additional information that may be used in the localization phase. The principle of radio map database creation is depicted in Figure 1.

#### 2.3.2. Localization Phase

During the localization phase, also called in some literature online phase, the position of the mobile device is estimated. The mobile device measures RSS values from all APs within range. These are sent to the localization server, which compares them to the data stored in the radio map database. Algorithms used for estimation of a position of the mobile device can be divided into two main groups-deterministic and probabilistic.

In the probabilistic (or statistical) framework the mobile device's position is modeled as a random vector. The location candidate γ is chosen if its posterior probability is the highest [23]. The decision rule uses Bayes' theorem:

(2) $$P\left(c_{i}|S\right)=\frac{P\left(S|c_{i}\right)P\left(c_{i}\right)}{P\left(S\right)}$$

where posterior probability *P*(*c_i_*∣*S*) is a function of likelihood *P*(*S*∣*c_i_*), prior probability *P*(*c_i_*) and observed evidence *P*(*S*) = Σ*P*(*S*∣*c_i_*)*P*(*c_i_*), vector *S* represents the observed RSS values during online phase and *c_i_* stands for *i*-th location candidate, *i.e.*, reference point (RP).

On the other hand deterministic algorithms are based on the assumption that RSS values at the receiver are not random and depend on the position of the mobile device [9]. Thus the position of a mobile device is estimated by searching for the highest similarity between the measurements from the device and the fingerprints stored in the radio map database. The position estimate is commonly computed using the estimator:

(3) $$\hat{x}=\frac{\sum_{i=1}^{M}\omega_{i}\cdot c_{i}}{\sum_{i=1}^{M}\omega_{i}}$$

where *ω_i_* is a non-negative weighting factor [9]. Weights can be calculated as the reciprocal of the distance between RSS vectors from the current measurements and radio map database. Usually the Euclidian distance is used, but different distance metrics are also possible [24].

The estimator of formula (3) that keeps the *K* largest weights and sets the others to zero is called the Weighted K-Nearest Neighbours (WKNN) method [10]. WKNN with all weights *ω_i_* = 1 is called the K-Nearest Neighbours (KNN) method. The simplest method, where *K* = 1, is called the Nearest Neighbour (NN) method [11]. In [9] it was found that WKNN and KNN methods perform better than the NN method, particularly when values of parameter *K* are 3 or 4.

In the literature it is shown that both probabilistic and deterministic algorithms are able to achieve similar results in terms of accuracy [23]. The advantage of the deterministic algorithms is that there is no need of an accurate statistical model to describe the signal characteristics in the environment. Therefore we decided to implement deterministic algorithms to the proposed modular positioning system.

## 3. Proposed Algorithm and Modular System

### 3.1. Modular Localization System

The proposed modular localization system is logically one level above the structure of standard localization systems. The components of a modular system have to be proposed according to the goals and requirements for such system.

The proposed system is an integrated set of components that provide localization services for its users. The proposed system should be able to provide services to multiple users and the service should be accessed simultaneously. The proposed localization system is centralized and thus a localization service is provisioned from one source called a localization server. It can be described as a network-based system with device assistance. This means that all computations are performed at the centralized localization server implemented on the network side of the system and the mobile device performs measurements when requested by the server. The system is fully autonomous, because it communicates using the existing infrastructure of telecommunications networks. Switching of the system mode (indoor or outdoor) is currently performed automatically based on data measured by mobile device. The process of switching is controlled by a modular localization algorithm (MLA).

**Figure 2.**
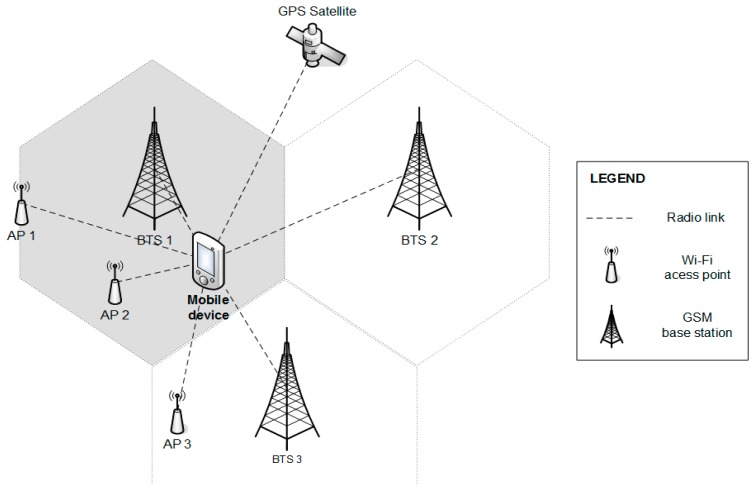
Example of an environment.

The proposed system is currently based on the assumption, that Global Positioning System (GPS), GSM and Wi-Fi together could allow ubiquitous positioning in an environment similar to that illustrated in Figure 2. Positioning modules based on these technologies are considered as basic localization modules of the system, since standard mobile phones contain the necessary hardware to exploit them. The system is open for implementation of other modules according to requirements, e.g., Bluetooth, Zig-Bee, *etc*.

The system can be divided into several components that communicate with each other and have their own responsibilities. The basic components are mobile devices (localized terminals), existing network infrastructures and the localization server. In terms of architecture, it is possible to describe the system from multiple perspectives that explain the importance and role of individual components such as:

- functional view,
- component view,
- technological view,
- user view.

These perspectives will be presented in order to comprehensively describe the modular localization system implementation. The functional view also contains the description of the novel modular localization algorithm.

#### 3.1.1. Functional View

The functional view represents the role and responsibilities of the various components of the system. These tasks can be divided into multiple layers, namely:

- *Presentation layer*—Provides visualization of the position of the user, allows him to control the localization of software, make requests and import radio map data.
- *Application logic layer*—Contains algorithms for position estimation, data processing and validation, security control and user request processing.
- *Service layer*—Provides information transfer between individual components.
- *Security layer*—Prevents unauthorized access, secures the transmission of sensitive data.
- *Management layer*—Monitors important changes in the system, records the errors and allows their analysis.

Localization system features can be divided into these layers for the mobile station, as well for the localization server. This division is shown in Table 1.

**Figure 3.**
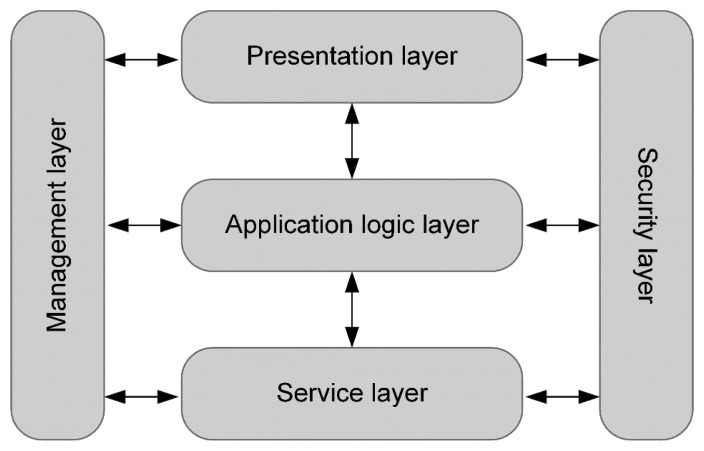
Functional layers in the modular localization system.

The individual layers are classified into three levels, where each layer communicates directly only with neighboring layers. Security and management layer are needed at every level and can communicate with any of the three layers. All layers in the system are shown in Figure 3.

**Figure 4.**
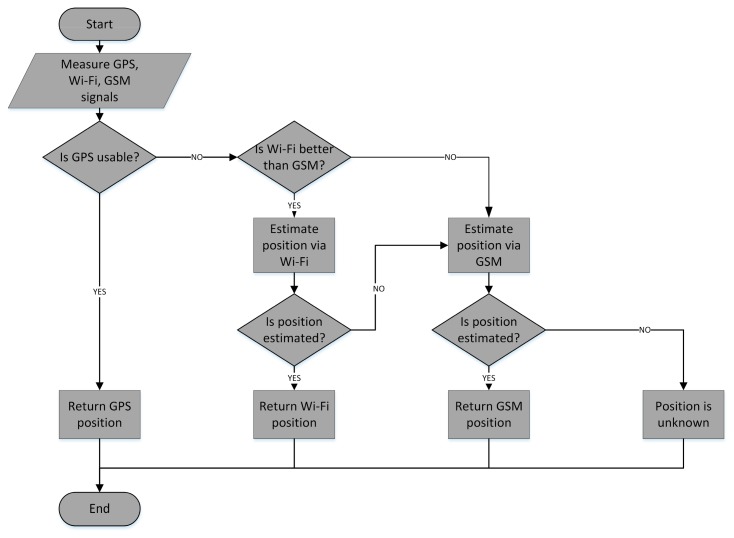
Flowchart of modular localization algorithm.

The application logic layer contains the original MLA algorithm. The algorithm is responsible for handling localization requests with all necessary signal information from GPS, GSM and Wi-Fi, selecting the most appropriate platform and returning the position. The details are depicted in the algorithm flowchart in Figure 4. The algorithm facilitates deterministic the fingerprinting localization framework, described in the previous section, together with the proposed map reduction algorithm.

In short, the MLA checks GPS availability and if the position can be determined, returns the position to the mobile device. However, GPS may not be available in scenarios involving missing GPS hardware, not enough visible satellites or indoor environments. In that case Wi-Fi or GSM measurements are used. The number of base stations with RSS 10 dBm above the minimum measurable signal level is calculated for the Wi-Fi network (*N_AP_*) and GSM network (*N_BTS_*). If *N_AP_* is greater than or equal to 3, Wi-Fi localization is selected. The threshold of three transmitters (APs or BTSs) was chosen, since signals from at least three transmitters are needed to clearly define position in 2D space. If that localization fails, e.g., similar radio map vectors are missing due to a non-mapped area or if *N_AP_* < 3, GSM localization is performed.

The RSS thresholds were set to −90 dBm for Wi-Fi signals and −103 dBm for GSM signals, since most devices provide minimum RSS values of −100 dBm and −113 dBm for Wi-Fi and GSM signals, respectively. This threshold was added based on previous results published in [25] which show that low RSS values have a negative impact on localization accuracy, caused by the fact that these values are more significantly affected by RSS fluctuations.

#### 3.1.2. Component View

The modular view presents the components of the system in terms of unique characteristics and interdependencies. Modularity enables later improvements and addition of features to the system in the future. Individual modules can extend the functionality of mobile stations, localization server or a localization system as a whole. The proposed modules relate to localization system as a whole and their roles are as follows:

- *GNSS module*—Localization module that utilizes one or more GNSS, initially GPS.
- *GSM module*—Localization module that utilizes GSM network.
- *Wi-Fi module*—Localization module that utilizes Wi-Fi network.
- *Communication module*—Data exchange and requirement transfer between the mobile station and the localization server.
- *Presentation module*—Display position of a user on a suitable map of the environment, through which he can determine what is in its surroundings.
- *Security module*—Security of communication between the mobile station and the localization server.
- *Application logic module*—User or mobile station request processing.

Modules should be substitutable and thus, for example when safety requirements change, the security module can be substituted by a more suitable one or a new localization module such as WiMAX or Bluetooth can be added.

#### 3.1.3. Technological View

The technological view provides an overview of the technologies and the standards by which the system is constructed. The list of proposed technologies for localization server and mobiles stations is given in Table 2. The list consists of technologies used in the system developed by the authors, and the use of different technologies can cause minor changes in the performance of the system from the complexity point of view.

Localization server software is built on technologies from Microsoft because of our extensive experience with their implementation, but also due to the presence of high quality tools and a vast user and programmer documentation. Data storage uses a SQL-based relational database system and thus enables advanced data analysis with other tools directly from the data of the localization system.

The mobile station software is built on the Android operating system, which allows better access to the hardware equipment of the mobile station compared to other mobile operating systems such as Apple iOS or Microsoft Windows Phone. Furthermore the Java programming language is up to certain level similar to C# language that has been used in the localization server.

The data transfer between a mobile station and the localization server is based on Hypertext Transfer Protocol (HTTP) and underlying TCP/IP protocol suite. The advantage of the HTTP protocol is in its wide use, therefore it is enabled on the communication networks, devices, operating systems and it passes network firewalls easily. Efficiency of the communication is achieved by Representational State Transfer (REST) principles used to expose localization server web services. The REST architecture represents a lightweight, computational and data transfer efficient alternative to the Simple Object Access Protocol (SOAP), which has been used for web services for years. The REST architecture saves computational resources on the mobile stations, where it is highly demanded as well as on the localization server.

Another benefit of the HTTP protocol is its integrated support for authentication and data encryption. Users are authenticated via basic HTTP authentication. Transmitted data are encrypted with Transport Layer Security (TLS) or Secure Sockets Layer (SSL) protocols.

#### 3.1.4. User View

User view represents the capabilities of the system from the user perspective. Users can be divided into groups, according to their role and permissions. Basic user groups are:

- *Standard user*—Uses the system to obtain localization information.
- *Installer*—Sets up the system before it can be used by standard users and administrators.
- *Administrator*—Monitors system operation, updates the system and solves problems.

A person can be member of more than one group, for instance if the system administrator needs to obtain localization information, he would become standard user as well. Each action in the localization system requires membership in certain set of groups as shown in Table 3 below.

There are necessary steps to set up and configure the localization systems before it can provide the localization service. Three phases of the localization system lifecycle have been identified, namely:

- *Installation*—Installer configures the localization server and creates one or more system administrators.
- *Initialization*—Administrator creates accounts for users of services and installer fills the database with auxiliary data for localization modules such as fingerprinting radio map.
- *Normal operation of the system*—Standard users utilize localization service, the administrator monitors statistics and resolves any problems in the system. Administrator also updates the database with new auxiliary data.

### 3.2. Map Reduction Algorithm

In a modular localization system the area where the positioning services will be provided is significantly larger, thus the size of radio map is increased. This may result in a significant increase of the time required for the localization server to estimate the position of a mobile device. Another reason to optimize the complexity of the positioning process is the ability of the localization server to provide responses to localization requests from a large number of devices.

The aforementioned facts were the motivation to modify basic algorithms in order to reduce the complexity of the positioning process. The idea of the proposed algorithm is to filter out vectors that contain measurements from the same transmitters as the mobile device detected during the positioning process. In this way we can determine which vectors are in the area of mobile device and use them in the position estimation process.

**Figure 5.**
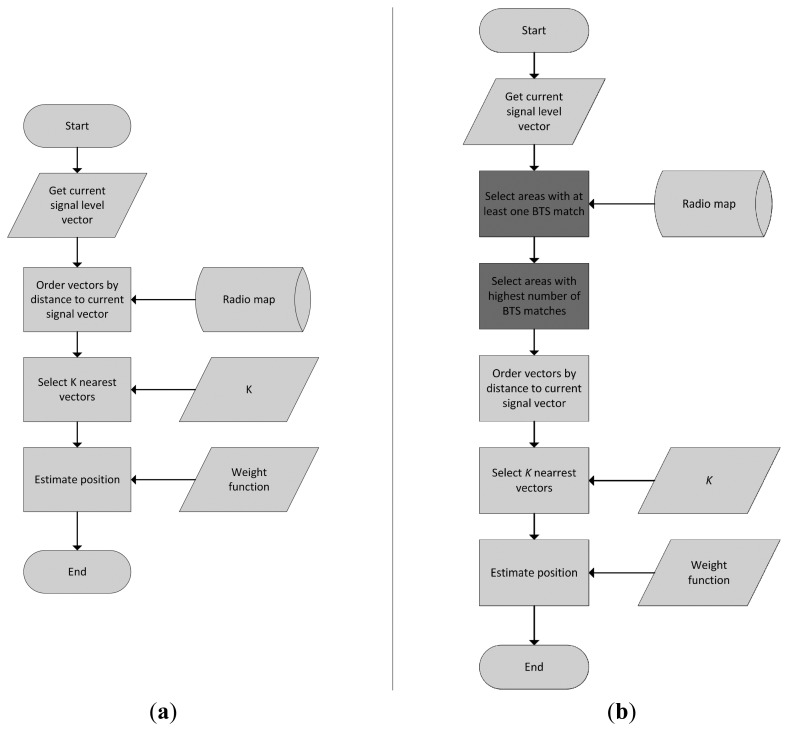
Flowcharts of the basic fingerprinting (**a**) and fingerprinting with 2-phase map reduction algorithm (**b**).

We called the proposed optimization algorithm as a 2-phase map reduction algorithm. In the first phase only relevant areas are selected from the radio map database. In the second phase reference points are selected from the relevant areas, thus the complexity of the system is reduced even more significantly. A flowchart of the proposed algorithm in comparison with the basic algorithm is depicted in Figure 5. The first phase of the proposed algorithm retrieves all vectors that match at least one transmitter with the measurement from mobile device. This is easily preformed via SQL language and the initial filtering reduces the radio map to the areas with least one matching transmitter in the range.

**Figure 6.**
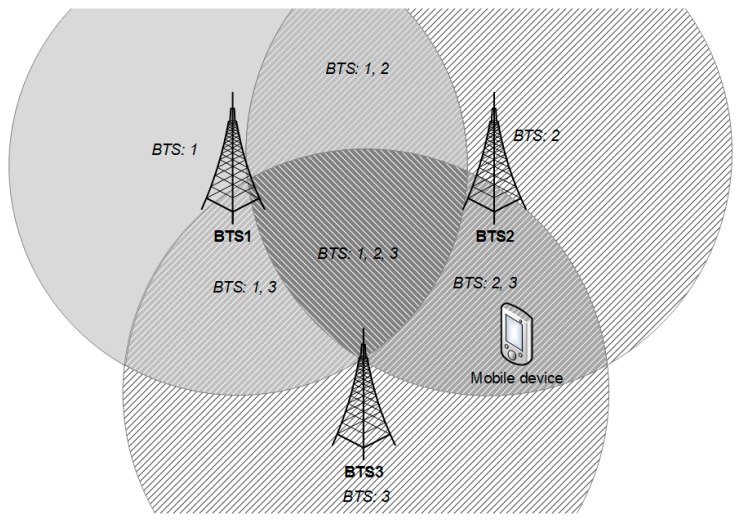
Phase 1 of map reduction algorithm.

Performance of this step can be improved by sound database administration practices, such as indexing or partitioning, which can pay off when the number of reference points in the map reaches millions. This reduction of the radio map is shown in the Figure 6. The algorithm will be described using a simple example, where only three transmitters (BTS1, BTS2 and BTS3) were detected during the offline phase and their signals are stored in the radio map. On the other hand, the mobile device detects signals from only two of them (BTS2 and BTS3) during the positioning phase.

In Figure 6 the situation when the mobile device did not detect signals from BTS1 during the positioning phase is depicted. Areas chosen by the first phase of the algorithm are marked with a diagonal pattern. It can be seen that areas where signals from BTS1 should be detected were also chosen. It can be stated that in the first phase of the algorithm, reference points were chosen from area covered by signals from at least one transmitter detected in the radio map, other than BTS1.

**Figure 7.**
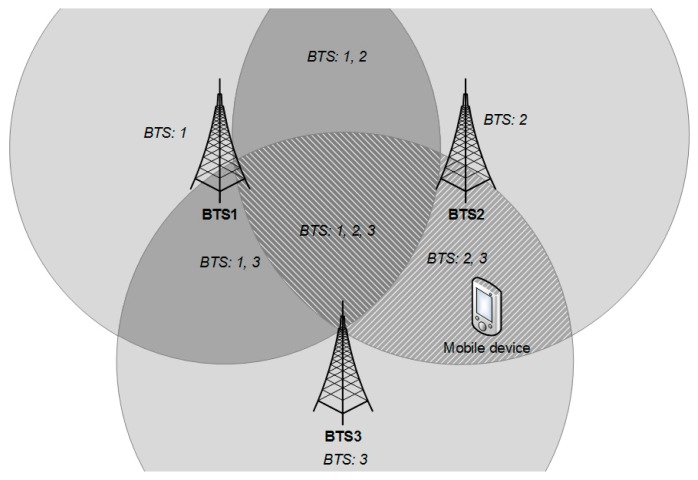
Phase 2 of map reduction algorithm.

In the second part of the proposed map reduction algorithm, it selects the most appropriate areas from the relevant areas chosen during the first stage as can be seen in Figure 7. This step is performed by ordering all areas by number of transmitters in the radio map vectors that match the transmitters detected by mobile device during the positioning phase.

As stated before, only signals from transmitters BTS2 and BTS3 were detected during the positioning phase. Therefore the highest number of matches is 2 and reference points were chosen only from two small areas, where signals from both transmitters can be detected. The highest number of matches is selected in order to handle power fluctuations which may cause the measurement vector from the mobile device to contain more or less transmitters that were detected at the reference points in the radio map database.

## 4. Testing Methodology and Setup

**Figure 8.**
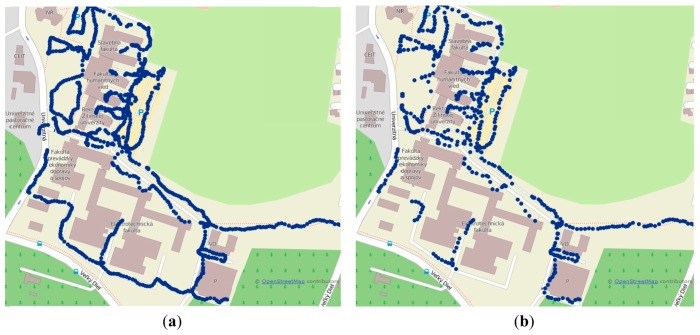
Outdoor radio maps for (**a**) GSM and (**b**) Wi-Fi displayed on Open Street Map.

Measurements were performed in the real world environment without any changes in the network infrastructure. Measurements were performed using implementation of the proposed modular localization system. The measurements were always coupled with the actual (*i.e.*, real) position coordinates either to create the radio map or during the positioning of mobile device, to compare estimated and actual positions. The actual position was taken from GPS in the outdoor environment. In the indoor environment actual position was entered manually in internal building coordinate system. Measurements in both phases were performed using Sony Xperia Arc with Android OS version 4.0 and the server software was installed on an Acer TravelMate 5744 equipped with Microsoft Windows 8.1. Both indoor and outdoor environments contained radio maps created from Wi-Fi as well as GSM. The RSS measurements in radio map use signal averaging to reduce fluctuations and improve robustness of the positioning system. Average RSS value was calculated as:

(4) $$\bar{\text{RSS}}=\frac{1}{N_{s}}\sum_{i=1}^{N_{S}}RSS_{i}$$

where *N_S_* is the number of samples, in the measurements set to 5, and *RSS_i_* represents *i*-th RSS sample. Radio maps were created during the evening hours, when the amount of traffic and pedestrians was lower, to achieve optimal results according to [26]. Measurements were performed in both indoor and outdoor environments. Radio maps for Wi-Fi and GSM in the outdoor environment can be seen in Figure 8.

Measurements in the outdoor environment were performed on the campus of University of Zilina; the size of the area was 780 × 470 m. The GSM radio map was created with 937 reference points. On average five BTS were detected per reference point. GSM measurements were performed in a single network operated by Telefonica Slovakia. On the other hand, the radio map created using Wi-Fi signals contained 574 reference points. This difference is given by the fact that reference points with less than three APs detected by the device were excluded from the radio map database. On average 15 APs were detected per reference point.

**Figure 9.**
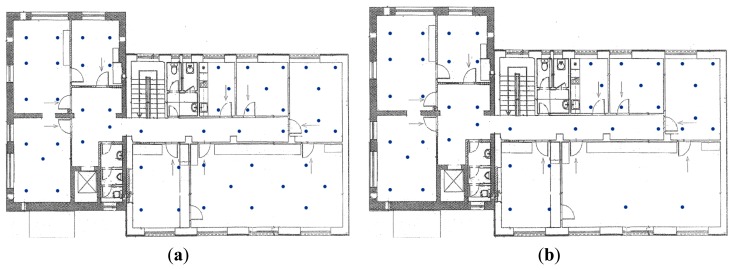
Indoor radio maps for (**a**) GSM and (**b**) Wi-Fi displayed on a plan of the building.

The indoor measurements were performed in an office building with brick walls located in Zilina. The size of the area was 15 × 8 m. The GSM radio map consists of 50 reference points. At reference points the average number of detected signals from BTS stations was 4.5. The spatial distribution of reference points in the indoor environment for both Wi-Fi and GSM is shown in Figure 9.

The radio map created using Wi-Fi consists of 43 reference points with average of more than 4 APs detected per reference point. Like the outdoor environment, the Wi-Fi radio map consists of a lower number of reference points compared to the GSM radio map, because a lower number of APs was in the range compared to the outdoor environment.

In the next section results achieved in the real world measurements will be presented and discussed. In order to do that we need to define the accuracy measures which were used to evaluate performance of the proposed positioning system. Accuracy is commonly expressed by the value of localization error, which is in fact a random variable and thus should be evaluated by statistical functions. Whereas accuracy usually refers to mean (*i.e.*, average) error values, the term precision is used for localization error-related properties.

The accuracy is commonly evaluated by Mean Square Error (MSE), Root Mean Square Error (RMSE), Circular Error Probability (CEP) and Cumulative Distribution Function (CDF). The MSE, in localization also referred to as Mean Distance Error (MDE), expresses average of the squares of localization error, then RMSE represents average localization error. Using 2D coordinates, the values can be calculated as:

(5) $$\text{MSE}=\frac{1}{N}\sum_{i=1}^{N}\left[{\left(x_{i}-{\hat{x}}_{i}\right)}^{2}+{\left(y_{i}-{\hat{y}}_{i}\right)}^{2}\right]$$

(6) $$\text{RMSE}=\sqrt{\text{MSE}}$$

where [*x_i_*; *y_i_*] are actual position coordinates of mobile device in *i*-th positioning step, [*x̂_i_*; *ŷ_i_*] are coordinates of estimated position, *M* is number of measurements. The lower the values of MSE and RMSE, the higher the accuracy of position estimates. These metrics can be used to evaluate 2D as well as 3D accuracy. In the result section, every RMSE value is accompanied with standard deviation *σ* of values in a form RMSE ± *σ*.

The CEP represents the radius of the circle where 50% of the estimated positions have an error lower than or equal to the accuracy value. The lower the value of CEP, the higher the accuracy of position estimates. This metric can be used to evaluate 2D accuracy. Similar circles can be defined for other statistically interesting values such as 67% (R67) or 95% (R95). R95 is one of the most important metrics to decide which of the two systems is more precise.

The CDF describes the probability that localization error will be smaller than some pivot value V. For N measurements in the set, it can be calculated as:

(7) $$\text{CDF}\left(V\right)=\frac{1}{N\left(\text{RMSE}<V\right)}$$

CDF is commonly expressed by a function graph from starting from *V* = 0 and ending where CDF(V) is close to 1. The metric can be used to evaluate both 2D and 3D accuracy.

Testing measurements were performed in two scenarios. The first scenario was proposed to evaluate impact of the proposed algorithm for map reduction. In this scenario outdoor positioning was performed with both Wi-Fi and GSM networks separately. During the measurements the localization accuracy and server response times were monitored.

The second scenario was proposed to evaluate performance of MLA algorithm in both indoor and outdoor environments. In the measurements localization accuracy of the modular localization system was monitored. In the measurements GPS module was not considered in the modular system. This should simulate the worst case scenario when GPS is not available and helps us to evaluate the performance of backup modules.

## 5. Achieved Results and Discussion

In the first scenario impact of the proposed 2-phase algorithm for map reduction on the performance of positioning based on fingerprinting framework was tested. The implemented algorithm was verified in the outdoor environment with both GSM and Wi-Fi radio maps. One hundred measurements were performed for each position estimator and network. The accuracy results before and after application of the proposed algorithm improvements are shown in Table 4.

From the results in the table it is obvious that map reduction improves the accuracy even if it was initially designed to only improve the performance. It can be seen that the mean localization error was reduced by 18 m for KNN, by 35 m for WKNN and approximately by 55 m for NN algorithm when GSM signals were used to estimate the position. On the other hand, improvement for Wi-Fi based positioning is even more significant. It can be seen that mean localization error was reduced from approximately 180 m to only 20 m, which is a significant improvement. This improvement is caused by the inaccuracy of the basic NN family algorithms, given by the fact that these algorithms do not consider the number of transmitters used to calculate the distance between RSS vectors. Thus basic algorithms can select RPs with only one detected transmitter with similar power, however in a completely different area, since other transmitters present at the given RP were not detected by the mobile device, the algorithms do not penalize this RP for missing transmitters and if the difference of RSS is relatively low, the position estimate is far from the real position. This problem of NN family algorithms emerges only when positioning is performed in large areas, since in small areas (e.g., buildings) the same transmitters are detected on all RPs.

The impact of the proposed 2-phase map reduction algorithm on performance of the localization algorithm is shown in Table 5. In the table *T* represents time of basic algorithm and *T_MR_* represents time after application of the map reduction algorithm. In the performance testing two kinds of different positioning requests, *i.e.*, valid requests and invalid requests, were used. Valid requests mean that measurement vector consists of RSS measurements from transmitters present in the radio map database and therefore the position of the mobile device can be estimated. On the other hand, invalid requests were generated with transmitters that were not present in the radio map database, meaning the measurements were performed in the area where the positioning service is not available and the position of a mobile device cannot be estimated.

It can be seen that map reduction significantly reduces the duration of the localization algorithm. The positive effect is more obvious in the Wi-Fi radio map, since Wi-Fi fingerprint vectors contain more transmitters thus the radio map is bigger. Another positive effect of map reduction is that it reduces the duration by three orders of magnitude for invalid localization requests.

The high values of standard deviation result from the fact that the application was running on a computer running on Windows OS and the computation time was affected by other processes running in the background, which causes high differences in computation times. It can be seen that the time needed to perform Wi-Fi-based positioning was significantly higher compared to the time needed to estimate the position using GSM signals. This was given by the fact that in Wi-Fi a higher number of transmitters was detected. Therefore more computations need to be performed to calculate the distances between RSS vectors.

In the last scenario, the positioning was performed in both indoor and outdoor environments at 10 evenly distributed positions. There were 10 localization requests executed at each position for all implemented positioning algorithms—NN, KNN and WKNN. Altogether there were 100 position estimations performed by each of the algorithms and environment. The achieved accuracy of the modular positioning system is shown in Tables 6 and 7 for indoor and outdoor environments, respectively.

**Figure 10.**
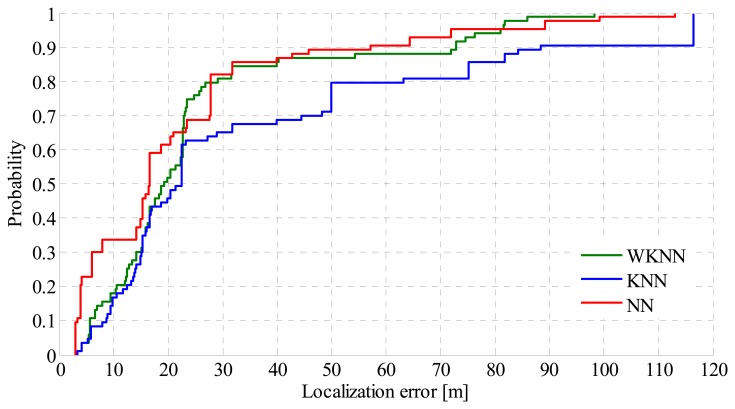
CDF of localization error in the outdoor environment.

From the achieved results it can be seen that positioning accuracy achieved by MLA positioning is slightly lower compared to the accuracy achieved by the Wi-Fi positioning with map reduction. However it is important to notice that Wi-Fi positioning is not available all the time and a low number of transmitters in the range can result in invalid positioning requests. Thus the accuracy of MLA is affected by GSM position estimates, which cannot achieve the accuracy of Wi-Fi positioning. In the outdoor environment the MLA selected Wi-Fi localization instead of GSM in 78% of cases, whereas GSM has been used only in 22% of cases. This is mostly caused by the signal coverage, because there were fewer positions with poor or no Wi-Fi coverage. For the better analysis of the achieved results CDF of localization error achieved by MLA in outdoor environment is shown in Figure 10.

From the figure it can be seen that NN algorithm achieved the best results and is able to estimate the position of mobile devices with an accuracy of less than 20 m in more than 60% of requests. It can also be seen that worst results were achieved by the KNN algorithm.

**Figure 11.**
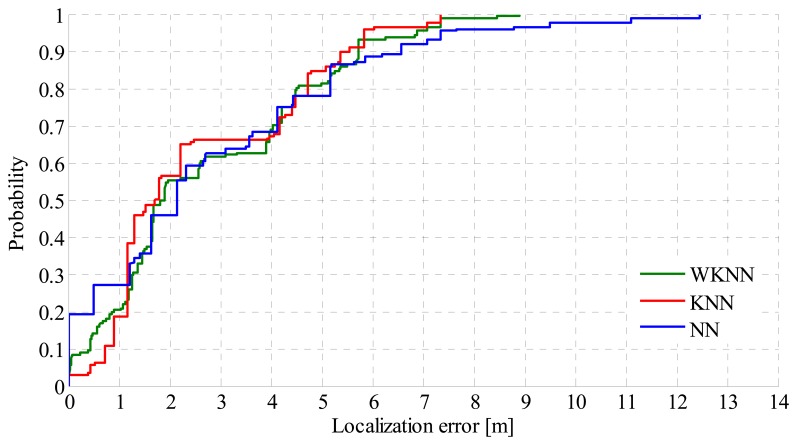
CDF of localization error in indoor environment.

In Table 7, the achieved results for an indoor environment are depicted. There it can be seen that the accuracy achieved with the MLA algorithm is much better compared to the outdoor environment. This is caused by the fact that radio signals are attenuated by the walls and furniture in the building, which results in the higher differences in RSS measured at reference points. In the indoor environment the MLA preferred Wi-Fi localization over GSM in 51% of cases. This is caused by the signal propagation in the environment, and it shows that in 49% of cases, there were less than three Wi-Fi APs with RSS greater than −90 dBm in the range of mobile device. Like the outdoor environment, the results achieved for the indoor environment are shown as CDF of localization error in Figure 11.

From the results shown in the figure it can be seen that, in contrast to the outdoor environment, in the indoor environment positioning using the KNN algorithm achieved best results. It can be seen that more than 55% of position estimates have errors lower than 2 m, which is a good result.

The differences between particular estimators are not significant; therefore we decided to implement NN in our modular localization system. The next very important fact is the computational capacity of particular estimators. The NN requires the lowest computational capacity therefore the decision seems to be optimal.

## 6. Conclusions and Future Work

In this paper a novel 2-phase map reduction algorithm and modular localization were proposed. Both algorithms were implemented in a localization system to evaluate their impact on the performance of the positioning system developed at the University of Zilina. Implemented algorithms were tested in real world conditions in both indoor and outdoor environments. From the achieved results it can be seen that proposed 2-phase map reduction algorithm reduced the complexity of the positioning process and also improved the accuracy of the position estimates. It has to be noted that the proposed algorithm allows detecting and ignoring false positioning requests, and thus can significantly reduce complexity.

A modular positioning systems that can utilize Wi-Fi and GSM signals was proposed and developed. The main contribution is the proposal of the modular localization algorithm, which is able to automatically switch between different localization modules. During the test, GPS positioning was disabled in the MLA algorithm, to determine performance of the positioning modules based on radio networks. The achieved mean localization error was 20 m in the outdoor environment and less than 3 m in the indoor environment. From the results it is obvious that positioning in outdoor environments still needs to be improved. However it must be noted that measurements were not performed in dense urban area, where a larger number of wireless transmitters and more buildings are present. This could allow slightly higher positioning accuracy. The proposed system can be used in transport for navigation purposes, since it can operate in both indoor and outdoor environments seamlessly.

In the future research the focus should be put mainly on optimization of the proposed positioning system, especially in outdoor environments, in order to provide more accurate results, even when GNSS-based positioning is not possible. We will also focus on development of an algorithm which will provide more precise switching between outdoor and indoor environments. Another interesting topic for the future research that can be performed on the proposed modular system is the impact of using different devices on the accuracy of the system.

## Figures and Tables

**Table 1.** Responsibilities of components in the proposed modular localization system.

**Layer**	**Mobile Station**	**Localization Server**
**Presentation**	Display user position	Display management interface
	Display other relevant data	
	Enter localization request	
	Control of map data collection	
	Configuration of application	

**Application logic**	Validation of input data Measurement and processing of localization data	Validation of input data
		Implementation of MLA and partial localization algorithms
		User request handling
		Error handling

**Service**	Data exchange with localization server Localization service calls	Data exchange with mobile stations
		Request transfer
		Insertion of data into database
		Reading of data from database

**Security**	Securing of sensitive data	Authentication
		Authorization
		Securing of sensitive data

**Management**	Notify localization server about errors	Make record of errors
		Make records of important changes in the system

**Table 2.** Technologies of localization server and mobile station.

**Localization Server**	
*Hardware*	Depends on performance requirements
*Operating system*	Microsoft Windows Server (latest)
*Web server*	Internet Information Services
*Database*	Microsoft SQL Server (latest)
*Software platform*	Microsoft .NET Framework
*Programming language*	C#

**Mobile Station**	

*Hardware*	Mobile phone with GNSS, GSM and Wi-Fi enabled hardware
*Operating system*	Android (2.3 and newer)
*Software platform*	Android SDK
*Programming language*	Java

**Table 3.** Actions in the localization system and user groups allowed performing them.

**Action (Permission)**	**Allowed Groups**
*Localize own person*	Standard users
*Localize someone else*	-
*Track own person*	Standard users
*Fill the database with necessary data*	Installer, Administrator
*Monitor the system*	Administrator
*Manage administrators*	Installer
*Manage users*	Administrator

**Table 4.** Impact of map reduction on localization accuracy.

**Network**	**Estimator**	**Basic Algorithm**	**With Map Reduction**

		**RMSE [m]**	**RMSE [m]**
**GSM**	*NN*	144.78 ± 78.15	89.67 ± 64.61
	*KNN*	110.44 ± 71.02	92.42 ± 50.90
	*WKNN*	120.63 ± 69.18	85.10 ± 61.45

**Wi-Fi**	*NN*	178.47 ± 47.57	18.20 ± 10.74
	*KNN*	181.24 ± 29.60	21.66 ± 13.18
	*WKNN*	181.42 ± 29.60	19.22 ± 8.70

**Table 5.** Impact of proposed map reduction algorithm on the response time of localization server.

**Metric**	**GSM**		**Wi-Fi**	
	
	***T* [s]**	***T****_MR_* **[s]**	***T* [s]**	***T****_MR_* **[s]**
Single valid request	0.68 ± 0.06	0.55 ± 0.17	4.31 ± 2.13	0.76 ± 0.60
10 parallel valid requests	3.44 ± 0.37	2.84 ± 0.95	23.14 ± 11.34	3.96 ± 3.33
100 parallel valid requests	30.76 ± 8.87	25.79 ± 10.81	289.86 ± 147.66	45.79 ± 41.90
Single invalid request	0.86 ± 0.14	0.006 ± 0.001	4.49 ± 2.37	0.014 ± 0.007
10 parallel invalid requests	4.32 ± 0.66	0.017 ± 0.006	24.15 ± 12.22	0.053 ± 0.033
100 parallel invalid requests	39.15 ± 12.27	0.220 ± 0.085	308.78 ± 178.89	0.505 ± 0.327

**Table 6.** Accuracy of the modular positioning system in an outdoor environment.

**Estimator**	**RMSE [m]**	**CEP [m]**	**67% [m]**	**95% [m]**
**NN**	22.58 ± 23.20	16.60	23.44	71.93
**KNN**	36.20 ± 33.82	22.37	31.68	116.52
**WKNN**	25.38 ± 22.04	19.30	22.76	81.06

**Table 7.** Accuracy of modular positioning system in the indoor environment.

**Estimator**	**RMSE [m]**	**CEP [m]**	**67% [m]**	**95% [m]**
**NN**	2.81 ± 2.65	2.15	3.58	7.35
**KNN**	2.58 ± 1.95	1.73	3.97	5.84
**WKNN**	2.74 ± 2.13	1.91	3.97	6.87
